# Policy Processes in Multisectoral Tobacco Control in India: The Role of Institutional Architecture, Political Engagement and Legal Interventions

**DOI:** 10.34172/ijhpm.2021.66

**Published:** 2021-07-14

**Authors:** Shinjini Mondal, Sara Van Belle, Upendra Bhojani, Susan Law, Antonia Maioni

**Affiliations:** ^1^Department of Family Medicine, McGill University, Montreal, QC, Canada.; ^2^Department of Public Health, Health Policy Unit, Institute of Tropical Medicine, Antwerp, Belgium.; ^3^Institute of Public Health, Bengaluru, Karnataka, India.; ^4^Institute for Better Health, Trillium Health Partners, Mississauga, ON, Canada.; ^5^Institute of Health Policy, Management and Evaluation, University of Toronto, Toronto, ON, Canada.; ^6^Department of Political Science, Faculty of Arts, McGill University, Montreal, QC, Canada.

**Keywords:** Policy Analysis, Multisectoral, Tobacco, Governance, India

## Abstract

**Background:** The development and implementation of health policy have become more overt in the era of Sustainable Development Goals, with expectations for greater inclusivity and comprehensiveness in addressing health holistically. Such challenges are more marked in low- and middle-income countries (LMICs), where policy contexts, actor interests and participation mechanisms are not always well-researched. In this analysis of a multisectoral policy, the Tobacco Control Program in India, our objective was to understand the processes involved in policy formulation and adoption, describing context, enablers, and key drivers, as well as highlight the challenges of policy.

**Methods:** We used a qualitative case study methodology, drawing on the health policy triangle, and a deliberative policy analysis approach. We conducted document review and in-depth interviews with diverse stakeholders (n = 17) and anlayzed the data thematically.

**Results:** The policy context was framed by national law in India, the signing of a global treaty, and the adoption of a dedicated national program. Key actors included the national Ministry of Health and Family Welfare (MoHFW), State Health Departments, technical support organizations, research organizations, non-governmental bodies, citizenry and media, engaged in collaborative and, at times, overlapping roles. Lobbying groups, in particular the tobacco industry, were strong opponents with negative implications for policy adoption. The state-level implementation relied on creating an enabling politico-administrative framework and providing institutional structure and resources to take concrete action.

**Conclusion:** Key drivers in this collaborative governance process were institutional mechanisms for collaboration, multi-level and effective cross-sectoral leadership, as well as political prioritization and social mobilization. A stronger legal framework, continued engagement, and action to address policy incoherence issues can lead to better uptake of multisectoral policies. As the impetus for multisectoral policy grows, research needs to map, understand stakeholders’ incentives and interests to engage with policy, and inform systems design for joint action.

## Background

**Key Messages**

** Implications for policy makers**

- A strong legal framework in tobacco control is essential for effective policy development and adoption to protect the health and human rights of the public.
- In countries with a federal structure such as India, the states play a key role in policy adoption and implementation, and hence attention must be paid to enabling a sub-national policy architecture, resources, structures and capacities.
- In a multisectoral policy, non-governmental, technical and research organizations can enable the formation of a supportive network architecture that plays a key role in evidence generation, advocacy, accountability and monitoring. The articulation of policy should open up spaces for societal participation and engagement.

** Implications for the public**

This research identified the important role played by a dedicated national program, mobilization of the political class and utilizing legal frameworks to safeguard the public interest in the tobacco control program in India. The leadership to drive the policy was played by the ministry and state department of health but supported organizations in stages of policy formulation and adaptation. Legal instruments like public interest litigation (PIL) and engaged citizenry and civic culture played a critical role in mobilizing and negating the tactics used by tobacco industry to promote tobacco products. Thus, citizen awareness, engagement and advocacy can play an important role in promoting and preserving the health of the populations.

Health policies play a central role in ensuring access, reducing inequities and improving health and well-being. The scope of these policies in today’s inter-connected world requires a more engaged and collaborative policy framework that moves beyond traditional silos so that political and bureaucratic institutions can engage and collaborate with one another, as well as with non-governmental organizations (NGOs), civic bodies and the citizenry.^1-3^ The objective of health policy today is to move from a sectoral to a multisectoral approach to enable work on the broader social, economic and political determinants of health, seeking coherence in policy-making and implementation.^4^

 Despite these objectives, policy processes remain poorly researched, especially in the context of low- and middle-income countries (LMICs).^5^ In the case of multisectoral policy in LMIC contexts, these problems are more acute as weaker institutional structures, and political fragmentation undermine the capacity to coordinate across multiple sectors.^6^ Understanding the role of the state, political institutions, actors and their interests, and the mechanisms in which they participate and yield power^7^ is imperative to advance our understanding of the role of collaborative governance arrangements in multisectoral policy implementation.^8^

 This study presents an analysis of a collaborative and multisectoral policy. We aim to generate in-depth insights into the process of national multisectoral policy formulation, adoption and adaptation at the state level, describing the context, the role of key actors in leading and influencing policy decisions, and detail the processes involved. Selecting tobacco control policy in India’s federal system as a case study, we explore how this policy was developed and how it progressed towards the implementation phase at the state and local level, explaining key drivers and challenges encountered. The Indian political system is a federal republic; both the National/Union government as well as sub-national/state governments enact laws. While “public health” is part of the constitutional mandate of states, tobacco as a commodity is a central matter. In fact, some of the regulation strategies used for tobacco (eg, food safety) fall on the “concurrent” list (that is, the responsibility of both national and state governments), so it is important to study actors and processes at all levels.

 The research questions guiding the study are: (*i*) What are the policies, directives, acts and laws guiding tobacco control in India? (*ii*) How did the context of tobacco control evolved at the national level and how was it adapted and adopted at the state level? (*iii*) What are the enablers, drivers and challenges in the Indian policy context? This analysis could be relevant to tobacco control policies elsewhere and more generally to multisectoral policy-making and implementation in other multisectoral scenarios and contexts.

###  Setting and Context

####  Tobacco Control in India

 India’s tobacco consumption has long ranked among the highest in the world.^9^ There are different forms of smoking products, mainly cigarettes and bidi (small hand-rolled cigarettes made of tobacco and wrapped in tendu leaf), as well as smokeless forms such as khaini (tobacco-lime mixture), gutkha (tobacco, lime and areca nut mixture) and betel quid with tobacco.^10^ According to the recent Global Adult Tobacco Survey (GATS), 42.4% of men and 14.2% of women use tobacco in one form or another.^9^ Apart from tobacco consumption, India is also the third-largest producer and exporter of tobacco, adding further contextual complexity.^11^

 Tobacco is a leading risk factor for major non-communicable diseases such as cancers, cardiovascular diseases and chronic respiratory illnesses, accounting for one in six of all non-communicable disease deaths worldwide.^12^ In 2010, smoking was estimated to have caused about 930 000 deaths,^13^ and smokeless tobacco caused 368 127 deaths in India.^14^ In addition, total economic costs attributed to tobacco use from all forms of diseases in the adult population (aged 35-69) was INR 1 104 500 crores (US$22.4 billion) in 2011.^15^ Direct costs of hospital care and treatments accounted for 16% of the total, whereas indirect costs of patient’s productivity lost due to premature death and loss of work and family income loss accounted for 86%.^15^ The threat from tobacco exposure has a wider impact that goes beyond health and extends to economic development, environment, and individual and social well-being. It also inhibits productive potential, as it increases the burden of morbidities, mortality and is also associated as a risk factor for chronic diseases making this a human rights issue as well.^16^

 Tobacco control policies and programs in India are comprehensive and promote multisectoral action both in their policy formulation and implementation.^17^ Tobacco control received attention in the early 1980s and 1990s, when national consultations on “Tobacco or Health” were held, which reinforced the need for protection from this health hazard. In 1995, based on a study commissioned by the central Ministry of Health, tobacco was identified as a “demerit” good with negative public health consequences. In 2003, a landmark national legislation was passed, the *Cigarettes and Other Tobacco Products Act*, 2003 (Prohibition of Advertisement and Regulation of Trade and Commerce, Production, Supply and Distribution) (known as COTPA). This provided the statutory mandate for action on tobacco control across the country. The evolution and enforcement of COTPA began at the same time as the emergence of the World Health Organization’s (WHO’s) *Framework Convention on Tobacco Control* (FCTC); India was the seventh signatory in 2004.^17^ This represents a unique case of ratifying an international treaty, marking a paradigm shift in regulatory strategy that affects both demand and supply sides.^18^

 These efforts gained more systemic momentum when the *National Tobacco Control Program* (NTCP) was introduced as a pilot phase in 2007-2008, followed by a gradual scale up during the 11th (2007-2012) and 12th (2012-2017) 5-year plans. Currently, the program is being implemented in all the 36 State and Union Territories.^19^ The creation of the NTCP had an incremental effect, as it provided a three-tier structure (national-state-district) and ensured much-needed adequate human resources and financial support across states and districts. It focuses on the implementation of COTPA 2003, conducting periodic training and information, education, and communication activities with stakeholders across institutions and departments, providing smoking cessation support and coordination to implement the program. It also created avenues for external oversight, monitoring and review of the program on a regular basis.^17^

 At the state level this study focusses on the Indian state of Karnataka. The state enacted a state-level legislation in 2001 known as the *Karnataka Prohibition of Smoking and Protection of Health of Non-Smokers Act*, 2001. The Act protected the non-smokers from the hazarads of passive smoking, especially in pubic spaces, discouraging promotion of advertisement for smoking and sale of cigarettes to persons under 18 years of age.^20^ There was not much time gap between the state and the national act in 2003, and the state-level momentum only began in 2004 after COTPA and with implementation of the NTCP. The state has also shown good results in enacting the law and implementing tobacco control policies at (sub) district levels.^21^ In addition, the GATS-I & II shows a significant decrease of 3.1 percentage points in smoking and smokeless tobacco and 5.4 percentage points in prevalence of tobacco use in Karnataka.^22^ The state also embodies the complexity of the tobacco environment, being the second largest producer of Flue-Cured Virginia tobacco, housing the largest cigarette manufacturer in the country and also manufacturing other forms of tobacco.^23^

 Together, COPTA and NTCP established the mechanisms for the promotion of coordination and cooperation between several ministries and their departments at the national and sub-national levels, and are recognized as an example for developing multisectoral policy in India.^24-26^

## Methods

 In order to trace the processes of policy formulation and adoption, we employed a single case study methodology with an embedded design.^27^ The embedded single-case study involves more than one level of the unit of analysis, in our case, the national and the state level. We used an established policy analysis approach (described below) to generate insights about the context, identifying factors influencing the policy-making, and detailing the policy processes in the case of tobacco control in India.

###  Policy Framework and Approach

 The health policy triangle framework^28^ serves as the foundation of this study’s design and the deliberative policy analysis approach^29^ guides our analysis of the data. The policy triangle framework is a well-established tool of policy analysis.^30^ We further detail on the processes of initiation, formulation and development of India’s tobacco-control policies, as well as to examine, step by step, the complex interactions amongst participants in the decision-making process.

- In context, we examined and described the use of tobacco, the burden of disease and social, structural and political factors that might have an influence on the policy.
- In content, we list the substance of documents and literature related to the selected policy.
- In actors, we identified persons and organizations playing a key role in the tobacco control program.
- In processes, using the Berlan et al, we discuss the approach to analyze the processes linking the phase of agenda-setting to the process of policy adoption through implementation, enabling the description of procedural, technical as well as political dimensions of policy design and adoption.

The deliberative policy analysis approach^29^ brings an understanding of the meaning of policies and laws as interpreted and applied in practice by the stakeholders.^31^ The approach is inclusive of the real-world plurality of interests and interpretation of stakeholders and networks. This approach reflects the practices and modes of current public sector governance, where collaborative working arrangements take into account experiences, values, understandings and beliefs. This is especially salient to tobacco control, which involves many diverse actors due to its multisectoral nature. We use this approach to construct a multi-faceted picture from the viewpoint of multiple actors, presenting a narrative grounded in and taking a more practice-oriented view.

###  Data Collection

 We collected two sets of data iteratively: in-depth interviews and documents for policy analysis.

 Document review: We sourced policy documents, acts, bills, government circulars/orders through web searches and also asked respondents to share key documents with our team as the first step. They were reviewed, collated and analyzed using a data extraction sheet. The database was prepared to track and ascertain the status and progress for implementation of tobacco control policy at the national and state (Karnataka) level. The document review also enabled the identification of key groups/departments or individual actors, who were followed up for an interview.

 In-depth interviews: The key informants were chosen mainly on the basis of their experience, relevance and their influential positions in relation to tobacco control policy formulation, development and implementation, either at the national state level (Karnataka) or in both. These include individuals involved in various capacities in the health system, civil-society organizations, high-level officials, experts and academics. These informants were all able to provide critical information, either as active participants in tobacco control policy development or as recognized subject experts. We selected respondents on the principle of maximum variation,^32^ to include and capture similarities and differences in perspectives across diverse stakeholders. We identified stakeholders through professional networks and colleagues and further requested them to identify and share information-rich respondents.^33^

 We conducted a total of 17 interviews, 8 at the national and 9 at the state level (see Table 1). Of these, 15 interviews were conducted in person, 1 by phone and 1 via Skype. In total, there were 6 female respondents and 11 male respondents. The interviews were conducted by the first author, SM, and were in English. We obtained written consent from all participants, and all interviews were digitally recorded. We developed a semi-structured interview guide that focused on the evolution and context of the initiation of policies; interviewees were asked to identify critical points/events in policy formulation and development and elicit challenges and opportunities at national and state levels. The average duration of interviews was one hour. Hand-written notes were taken, and summaries of the interviews were prepared to capture immediate impressions and reflections. Each audio-recorded interview was transcribed verbatim by the lead author and a research assistant, and the lead author was responsible for quality checks of the transcribed text. Each respondent was classified into a broad descriptor categorization to maintain anonymity and hence no organizational affiliations or socio-demographic information is shared.

**Table 1:** Types of Interview Respondents, Organization, and Gender

**Category of Respondents/Organization Affiliation**	**National/State**	**Gender **
Technical expert	State	Male
NGO member	State	Female
Academic organization	National	Male
NGO member	State	Male
Advocacy organization	National	Female
Advocacy organization	National	Female
Technical organization	National	Male
NGO member	State	Male
Health department	State	Female
Health department	State	Male
Education department	State	Male
Academic organization	National	Female
Legal expert	National	Male
Police department	State	Male
Health department	State	Male
Technical organization	National	Male
Technical organization	National	Female

Abbreviation: NGO, non-governmental organization.

###  Data Analysis and Synthesis

 We started with compiling and reading all the transcripts to familiarize ourselves with the data, and to reflect on the notes from each of the interviews. We undertook a thematic coding approach which included both inductive and deductive approaches.^34,35^ We developed a codebook through an iterative process of initial manual coding of transcripts. The coded texts were organized into themes, such as policy context, process, critical drivers, successes, challenges and suggestions for strengthening the program (Table S1, Supplementary file 1). Each of the themes was reviewed and studied to build a coherent narration and to note differences in perception and meaning expressed by the interviewees. Efforts were made to triangulate information from both the data sources.

## Results

 We present our findings in the framework categories of context, content, actors and process as identified by the policy triangle.^28^ The context is described in the settings and context section above.

###  Policy Content

**Figure 1:**
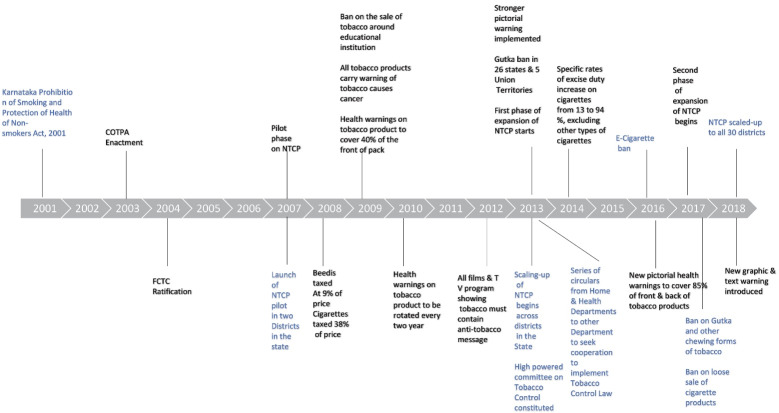
Timeline Indicating Key Policy Content Development at the National (Black) and State (Blue) Level. Abbreviations: COTPA, Cigarettes and Other Tobacco Products Act; NTCP, National Tobacco Control Program; FCTC, Framework Convention on Tobacco Control.

 We reviewed policies, acts, laws, government orders and circulars at the national and state level. We summarize the key points below in a timeline in Figure 1. The details are annexed in Tables S2 and S3.

 Throughout India, COTPA 2003 remains the principal law providing guidance on restrictions regarding smoking in public places: prohibition on advertising, promotion and sponsorship; regulation of sales to minors; packaging and labelling; and enforcement and penalties. However, legal provisions against tobacco started as early as the 1975 *Cigarette Act*. The regulation of tobacco was addressed under the national 1940 *Drugs and Cosmetics Act*, which banned the manufacture and sale of toothpaste and toothpowders containing tobacco. In 2000, the Indian government prohibited direct or indirect production, sale or consumption of cigarettes and other tobacco products by amending the *Cable Television Networks (Regulation) Act *of 1994. The *Food Safety and Standards (prohibition and restriction on sales) Regulation 2011*, prohibited tobacco and nicotine from being used in any food products, and was used by several states to ban the manufacture, distribution, and sale of “gutka” or “pan masala” (a chewing form of tobacco).

 The main policy document for the implementation of tobacco control measures is the *National Tobacco Control Program Operational Guidelines* of 2013 and 2015, which provides guidance for training, information, education and communication activities, the monitoring of tobacco control laws, coordination with other institutions, and setting up cessation facilities. It also provides the financial support for these activities and supports human resources to operationalize the program at national, state and district levels.

 Karnataka was one of the first Indian states to ban e-cigarettes in 2016. It prohibited the sale, manufacture, distribution, trade and advertisements of Electronic Nicotine Delivery System, noting that nicotine in food products and consumption is banned under the *Food Safety and Standard Act* of 2006 and *Food Safety and Standards (Prohibition and Restriction on Sales) Regulation 2011*. Circulars and follow-up orders were issued by the Deputy Secretary to the Health and Family Welfare Department to ensure that senior state bureaucrats and district functionaries would be able to implement this ban.

###  Actors

**Figure 2:**
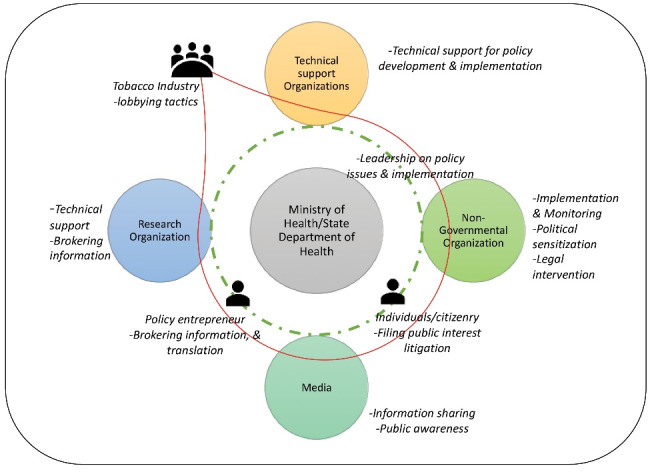
Key Actors and Their Roles in the Policy Process for Tobacco Control Policy. All actors, except for the tobacco industry (depicted by red colour), influenced the policy adoption in a positive manner.

 The tobacco control program’s development involved the participation of diverse individuals and organizations, where some were mandated to realize the program and others were motivated to reduce the tobacco use. Overall, we found an interdependent, reciprocal and networked relationship between these organizations and individuals to limit the use of tobacco. These organizations did not work in silos and often had a supplementary and complementary roles to each other; we further explain this in the processes section. The main actors, their roles and activities are depicted in Table 2. Figure 2 illustrates key actors and their roles in the policy process for tobacco control policy. All actors play interconnected, reciprocal and overlapping roles, except for the tobacco industry (depicted by red colour), which influenced the policy adoption in a negative manner.

**Table 2:** Key Actors, Their Roles and Activities in the Tobacco Control Program

**Actors**	**Role**	**Responsibilities**
Ministry of Health and Family Welfare/State Department of Health and Family welfare	Leadership on policy issues and implementation	Convening of expert groups/meetings/advisory groups Assessing policy alternatives Drafting/issuing policy guidelines Implementation guidance and support Seeking support from associated ministries/departments
Technical support organizations (global and international scientific organization)	Technical support for policy development and implementation	Support Ministries of health at the national/state level Drawing lessons from the global/regional experience Compiling research evidence to support policy Organizing expert consultation Implementation capacity building
Research organizations (national and state)	Technical support Brokering information	Conducting contextual research (estimating burden of disease, implementation research, policy evaluation) Sharing/translating scientific information into a common language
NGOs (global, national and state level)	Implementation and Monitoring Political sensitization Legal support Advocacy	Monitoring the global activity of the tobacco industry Monitoring tobacco industry activity during negotiations Monitoring positions of delegations during negotiations Filing PILs Creating political mobilization
Media (national and state)	Information sharing Public awareness	Generating awareness and creating public opinion
Policy entrepreneurs (national and state)	Brokering information Knowledge translation	Organizing information sessions for policy-makers Channeling information to governments Providing alternate policy mediations based on evidence
Individuals/citizenry	Personal legal interventions	Filing PILs
Tobacco industry	Interest group-lobbying	Push for pro-tobacco products, newer products like e-cigarettes and vaping devices Creating alternative narratives/doubts by overplaying economic and livelihood significance Negating implementation of tobacco control laws by marketing and advertisement Filing multiple legal cases across the country

Abbreviations: NGO, non-governmental organization; PILs, public interest litigations.

###  Processes

 We contextualize the Berlan et alapproach to analyze the processes in a non-linear manner, capturing the back and forth process of endorsement and rejection.^36^ The most important steps are: the role of research and evidence in the generation of policy alternatives; deliberations, consultation and the role of expert opinion; political sensitization and legal interventions; lobbying to influence policy decisions; and state adoption and implementation. These are key steps in the process but are not necessarily sequential and may take place concurrently, influencing and overlapping one other. While these policy processes are fluid and continuously adapting and modifying, the tracing of these steps allows for the identification of the processes and activities engaged at each stage and provides analytical clarity to the reader.

####  Generation of Policy Alternatives: The Role of Research and Evidence

 The presence of evidence-based knowledge and the ability to adapt and synthesize contextual knowledge was expressed as the foundation of the tobacco control program in India. The generation of policy alternatives was taken up by research and technical support organizations through two interrelated processes: first, by conducting policy relevant primary research; and second, by sharing available best practices from across the globe. According to a state-level technical expert:

*“So here there was an academic guy or an institute like Institute X that was actually telling us what works and what does not work, and trying to influence the policy. We are trying to make up a policy, and they share that, according to this certain piece of research, this intervention doesn’t work, so please don’t waste time on this. That’s the way we actually envisaged the synergies, that we are going to use out of academia to change policy” *(State level technical expert).

 These research and technical organizations worked in complementarity by identifying the needs of the policy community and providing them with the best available knowledge and link to technical experts to discuss the alternatives available. However, apart from the global best practices, context-specific research allowed for the adoption of relevant practices in the NTCP. For example, conducting grouped randomized control trials to observe differences between schools receiving tobacco control intervention and commissioning studies on the economic impact of tobacco enabled the Ministry of Health and Family Welfare (MoHFW) to scale up its evidence base. Similar research support was available at the state level, where initial studies focused on descriptive and explanatory studies to understand the nature of the tobacco control challenges and how implementation can be better adapted and provide essential feedback.

 The policy-relevant research of these research organizations, took a “*360-degree approach,”* including studies of individual, structural and socio-cultural determinants associated with tobacco use, especially the attractiveness of smokeless forms of tobacco to young people, and socio-cultural traditions related to chewing tobacco. Specific threads of research also focused on the role and influence of the tobacco industry, and aspects of conflict of interest within government related to tobacco control. Such contextually relevant scientific evidence contributed toward informed decision-making.

####  Deliberations and Consultations: Discussions and Expert Opinions

 During the process of deliberation and consultation, the MoHFW and State Health Departments provided the leadership, and the technical organizations also joined in to provide expert opinions. The enactment of COTPA in 2003 and the guidance for implementation of the NTCP and FCTC stressed the importance of engaging multiple stakeholders in order to seek cooperation and coordination from multiple sectors and organizations, to seek a system-wide, comprehensive effort. These mandates provided an “invited space”^37^ for multi-stakeholder consultation and engagement. Examples of a national inter-ministerial group, a state-level high-powered committee on tobacco control, policy level deliberation forums and consultation with the Ministries of Finance, Labour and Welfare, Commerce and Information were quoted by stakeholders as ways of aligning mandates of different departments for tobacco control and promoting an multisectoral approach. However, these forums to engage and get agreement between ministries required considerable coordinating efforts and regular monitoring to ensure follow-through on decisions. One of the core challenges remained to align the mandates of the different ministries; for example ministry of health was mandated to fight against tobacco, but the agricultural ministry promoted tobacco cultivation as a cash crop, which affected participation, consensus building and the unified work for tobacco control.

 The MoHFW convened and hosted numerous other expert committee meetings during the course of the tobacco journey, and these were supplemented by consultations organized by the technical support organizations towards supporting policy development, bringing together a network of experts, civil society organizations and collating and presenting research evidence from international settings. One of the national-level respondents shared:

*“We bring the best technical support to the table. If we don’t have the expertise within, we bring the best experts to the table. If we don’t have the best manuals in place, we bring down manuals, we create; we get the best heads around the table. Most of the committees are built of local experts and if required, global experts wherever necessary” *(National-level technical expert).

 These consultations also served as a platform to translate complex research language into a commonly understood narrative. For example, during the expert committee meetings, members of civil society and research experts translated scientific evidence to help policy communities to understand and utilize it by explaining them in lay terms and weighing their opinion on the evidence shared, and thus linking ‘evidence to policy translation.’ These consultations and deliberation with multiple stakeholders across sectors and institutions enabled broad-based discussions, evidence translation and policy diffusion.

####  The Mainstay: Political Sensitization and Legal Intervention

 The advocacy efforts in tobacco control have focused on mobilizing legal and political components of policy and utilizing them as a mechanism to generate momentum for action. Political sensitization was aimed at generating momentum to promote leadership for policy initiatives and advocacy organizations and the NGOs actively engaged in the process. This involved engaging and sharing evidence with elected leaders and bureaucrats like political leaders, members of parliament, ministers and deputy chief ministers, including secretaries across different ministries. These organizations also enabled the members of parliament to answer questions related to tobacco, thus gaining their trust and working with them mutually. The role of policy entrepreneur was also highlighted in generating political momentum, as these entrepreneurs had sound scientific knowledge and were respected as experts on the subject matter, and engaged with the political party representatives. This allowed for the advancement of agendas in parliamentary sessions to draw attention, build political momentum and action on the issues.

 The aspect of legal mediation, using public interest litigation (PIL), was important in the Indian context, as the program faced resistance from the tobacco industry and required legal safeguards to ensure the public interest. The legal guidance is provided by COTPA, a national law; however, it was the NTCP that provided the position for legal advisors at the national and state level to systematically engage with legal cases. This allocated necessary human and intellectual capital, trained in legal practice, to counter the legal aspects of industry interference.

 Support from individuals, non-governmental and civil society represented a key effort in legal interventions. PILs questioned the intent of governmental decisions, aspects of exploitation of deprived groups and argued for the promotion of a health and human rights approach. Some of the key PILs at the national level focused on the enforcement of graphic warnings on tobacco product packaging; at the state level, PILs challenged the participation of ministerial bodies in industry-sponsored events and selling tobacco to minors or in the vicinity of educational institutions. The Indian judicial system was described as very supportive of tobacco control measures by the respondent groups. Both the high courts and the Supreme Court of India provided crucial judgements that banned chewing forms of tobacco, tobacco industry sponsorship of government meetings and maintained 85% pictorial warnings on the tobacco products packaging. Some stakeholders described the judiciary decisions as vital in preventing the industry to go *“forum shopping*,” where they adopt the practice of getting their cases heard in a particular court that is likely to provide a favourable judgement.

 “*Research forms the base of it and, of course, articulating that research well to the policy-maker. But eventually, when the push comes to shove, it’s always judiciary because the industry always goes to court and they do forum shopping, they go all over the country” *(National level advocacy organization).

 The judicial rulings were seen as “*providing a safe gateway*” to implement tobacco control measures, particularly with the legal foundation of COTPA. However, these legal interventions, like PILs, were often contested, dragging on and at times overturned by legal interventions by the tobacco industry. The example of section 7 of COTPA was quoted as an example, which provides the pictorial health warning on the tobacco products packaging. The tobacco industry representatives constantly challenged the governmental decision to implement strong pictorial warning, and this led to many back-and-forth legal cases lasting over a decade. Thus, in order to support such legal battles required legal advocates to be vigilant and a constant engagement to maintain the legal decisions in favour of the public interest. The role of media was quoted to be very supportive and important in highlighting and informing the general public about current advances in the program. Media engagement was defined to be systemic where national media houses and local vernacular media covered the legal stories, keeping the public interest alive and garnering support through mass media. This media engagement was often termed as ‘media advocacy’ and was cited as an efficient investment, which had a wider outreach with limited monetary resources. The political sensitization and legal interventions were essential additional safeguard mechanisms for efforts of the tobacco control program.

####  Tobacco Industry Lobbying to Influence Policy Decisions 

 The tobacco control program in the country faced the challenge of countering tobacco industry measures, as tobacco has a revenue generation component and drives the industry to maintain its proceeds. The tobacco industry engaged in lobbying campaigns, influencing the policy processes and the stakeholders involved at both state and national levels. Instances of both ‘inside’ and ‘outside’ lobbying were mentioned by the respondents. Inside lobbying included holding positions on executive committees or agencies by individuals or groups having an interest in tobacco production or with tobacco industries. Outside lobbying included sponsoring events by tobacco industries, organizing and coordinating the tobacco farmers’ movement against tobacco control to influence policy-makers and using alternate means to advertise tobacco products without branding to counter advertisement laws. The industry has also tried to frame tobacco control as being “anti-farmer” – since tobacco cultivation as their source of livelihood.

 Although Karnataka is a tobacco-cultivator state, we did find examples of organizations and research institutes that attempted to negated these accusations. They worked on identifying a combination of crops with high returns, as well as simultaneously engaging with farmers to enable them to shift from tobacco cultivation to alternate crops. These organizations presented the farmer narrative in terms of what would be required for transitioning to such alternatives:

*“ So the idea is to encourage farmers’ to understand their narratives and what are their demands. Many farmers want. for example, a better water supply, if you want them to switch because many other crops are water-intensive. They want some sort of subsidies to grow (other crops)” *(State level NGO member).

 Presenting alternate narratives and sharing the details of farmers’ needs, along with the advocacy efforts of civil society groups, helped counteract the tobacco industry’s argument that farmers would be harmed by tobacco control laws. The industry was described as resourceful, intuitive, and highly innovative in promoting new ways to either increase the uptake of existing tobacco products or introduce newer tobacco products. It also played a key role in the promotion, advertisement, and distribution of tobacco and tobacco-related products.

####  Guiding Implementation: State Adoption and Moving Into Action

 Implementation at the state level was guided by the operational guidelines set under NTCP and spearheaded by the State Health Department, especially the State Anti-Tobacco Cell. In the initial phase, action at the state level centred on piloting implementation in two selected districts cells. The effort was funded by the MoHFW and subsequently co-financed by the state government. Expansion of NTCP began in 2013-2014, and by the end of 2017-2018, it was expanded to all 30 districts in the state. The process was gradual, as actors had to be sensitized first, and specific structures had to be established.

*“Initially we (have) sensitized the key persons of the stakeholders and the chairman, and they have issued few circulars saying that formation of district tobacco cell, formation of state cell at the state level and formation of squads (multisectoral team to conduct enforcement of the law), all these things, it came through the state level directions and the district level directions”* (State level health official).

 During the early phases of implementation, technical support organizations worked directly with this cell in providing technical support through human resources or by providing capacity-building support, working on both fronts of management and technical training. The technical support staff worked with the health department in creating a ‘politico-administrative framework’ to sustain the program at the state level, as they built necessary institutional structures and mechanisms.

 The other key ‘institutional mechanism’ at the state level that enabled decisions and resolutions to activate tobacco control was the establishment of a what was known as the “High-Powered Committee on Tobacco.” The membership comprised all the principal secretaries of key departments, civil society and scientific representation. This committee issued orders to the departments of health, education, police, and urban development, underlining the importance of joint efforts required for tobacco control. This, in turn, was followed by respective departments issuing circulars to their own divisions, asking them to support the display of no-smoking signages and creating smoke-free environments. The committee encouraged action *“by creating structural shifts and forming a platform*” to apply for the “*three Rs-Report, Review & Reinforce through a higher level body*.” This also acted as a mechanism to garner the support of non-health departments to take action.

 Sensitization and uptake of policy were initiated through numerous training programs and workshops. These aimed at state and district administrative levels across various departments, facilitating the execution of institutionalized enforcement mechanisms, such as district and state-level coordination committee meetings, and the formation of tobacco enforcement teams at the district level. The aim was to ensure support across stakeholders from the highest level to the enforcement officers. The sensitization involved information on the burden of tobacco use, videos of tobacco victims, briefing on COTPA sections, the number of fines to be collected, and the role of each department/stakeholder. The engagement of local media to increase awareness about tobacco control and report on key enforcement activities was also highlighted as an important outreach media strategy by respondents.

 At the state level, NGOs and advocacy organizations played an important role in enabling the implementation environment. This required nudging of various departments to issue government orders with regard to participation, coordination and implementation. These state orders acted as official reminders and highlighted the role of each department in the program, and facilitated coherent training, sensitization and hiring or allocation of designated human resources for the program across all departments:

*“Because as a mandate, they are supposed to train and sensitize different stakeholders, but they can’t go each department and get the orders out. They can only work what’s there, as they come from the health department, they can only ensure that their seniors issue strong departmental orders. Even with a nodal officer (present for the program), he cannot go to secretary health and say sir, please issue this order, that also civil society has to do” *(State-level administrator).

 NGOs, research and technical support organizations also supported the State Health Department by providing assistance in compliance assessment surveys, and aiding in monitoring, evaluation and feedback on the ongoing implementation activities in the field. The stakeholders engaged during the expansion described this as a challenging start, as it was difficult to gain priority for a new program that required cooperation and support across many departments. Stakeholder sensitization process of senior officers from state and district level required constant and active engagement.

## Discussion

 The analysis of the development and adoption of tobacco control policies in India reveals a process of collaborative action among most of the actors identified in this study. The tobacco control issue, characterized by the need for interdependency and interaction, depended on strong coordination amongst all players as a pre-condition. At the same time, working towards preventable and avoidable deaths provided the incentive to work together. This collaborative action was also catalyzed by key drivers. In a “Collaborative Governance” framework, these are considered the drivers of policy process initiation that can set the direction for collaborative practices, and as the necessary conditions for the impetus and success of collaboration.^38,39^ Our study found the key drivers for this process to be the institutional mechanisms for collaboration, multi-level cross-sectoral leadership, and political motivation and mobilization.

###  Institutional Mechanisms for Collaboration

 The provision of institutional mechanisms under NTCP facilitated coordination and collaboration across different ministries and at the state level enabled implementation support through a three-tier structure and necessary financial and human resources. The other institutional arrangements to aid decision-making were the formation of an inter-ministerial group at the national level and a high-powered committee at the state level. At the national level, this group worked towards bringing alignment between ministries with different mandates in tobacco control, such as health, commerce, and agriculture. At the state level in Karnataka, this committee formed a key decision-making platform, led by the principal secretary of the state, and participation from all other departments. This committee served to review, coordinate, problem-solve, and issue key directives/circulars for effective implementation.

 However, across national and state levels, concerns were expressed about the functionality of these coordination committees, which often needed nudging and steering to transform words “*on paper*” into real action. The supportive role of advocacy and policy entrepreneurs was noteworthy in making these mechanisms truly functional.

###  Multi-level, Cross-sectoral Leadership 

 The tobacco control program demonstrated leadership initiatives across national and state governments, ministries and policy sectors, researchers and policy advocates. The supportive and facilitative roles played by these actors enabled the functioning of institutional platforms and provided stewardship. The national level stakeholders noted the MoHFW’s key role in providing the leadership on policy issues. The ministry took a steering role in examining evidence for policy and legislation by forming several technical advisory committees to provide guidance. At an individual level, ministers at the national and state level took crucial decisions regarding nationwide cigarette package warnings and banning the use of “gutka” at the state level. In administrative roles, the commissioners and respective heads of the departments became local champions in participating and leading efforts for policy adoption.

 While it was important for health departments to lead these efforts, it was also necessary to form alliances and build leadership within other engaged sectors for the uptake of policy and implementation. As one state level respondent remarked, “*that is what takes along the program*,” highlighting the importance of leadership across sectors. At the state level, collective action required building collective leadership across bureaucrats, police officers, health officers, and NGOs.

 This leadership was also evident within the medical community, as medical doctors trained as public health professionals, researchers and cancer specialists, engaged with the process of evidence generation, advocacy for policy uptake and furthering and refining implementation. Their role was seen as creating synergies between realms of policy and science. They utilized their positions of power to bring leadership and influence to the issue of tobacco control.

###  Political Attention and Mobilization

 The tobacco control program garnered considerable political attention as politicians across political parties, whether in government or opposition, took a consistent interest. Political pressure was evident in press conferences, civil society involvement, as well as in asking key questions and debating issues in the parliament. At times, politicians were personally motivated by the issue of tobacco claiming lives or were catalyzed by pressure from advocacy organizations. However, the political commitment towards tobacco control was not always positive. For example, respondents from civil society, highlighting the example of the bidi industry at the state level, detailed that some of the ownership of this industry is shared by politicians, and it was not necessarily always easy to gain political momentum on tobacco control in the initial years of COTPA.

 The political support also remains contested, especially where cabinet decisions were required. The other common problem noted with political mobilization was the need for constant engagement and adaptation to changes in political regime, such as identifying the political advocate who would be the ‘best buys’ for the ongoing support of the tobacco control initiative. However, despite these limitations, the political environment has been largely supportive by providing measures such as a dedicated national program, enhanced taxation on tobacco products, and the gutka ban at the state level.

 Apart from these critical drivers, the overall dynamics of collaboration centre around the process of actor engagement, motivation and the capacity for joint action.^38,39^ In tobacco control, this collaboration was built on the creation of trust, shared understanding, and commitment to the process. In this case, the “invited spaces”^37^ for engagement were provided through national and global policy mandates, allowing partnerships among organizations, stakeholders, departments and sectors for joint action. The roles and responsibilities played by these actors were readily accepted as being ‘credible’ and ‘legitimate,’ and well-respected as each actor was specialized in their own domains of technical support, research and advocacy in health in India. The collaborative thread binding or sentiment motivating a joint call for action was the narrative of “tobacco kills,” and that tobacco-related deaths are preventable. It served as a very strong motivation to work together towards promoting health and human rights as a counterweight to arguments about the economic gains from addictive products.^40,41^ Hence, collaboration against tobacco as a common enemy causing public harm provided mutual benefit and gain, sustaining joint action. This case of a collaborative, multisectoral policy clearly identifies that the terrain of health policy analysis has become more networked, and represented by plural stakeholders. Hence, it becomes essential to map these wide ranges of stakeholders and interest groups to understand their perspectives and interests to engage with the policy.

###  Challenges in Tobacco Control and Future Considerations

 Despite the fact that the collaborative process builds on finding common ground, objectives and trustworthy relationships,^42^ it is not free from conflict.^43^ The pre-condition remains compatible and interdependent interests,^38^ and the most common challenge remains with the process of collaboration and substantive problem-solving.^44^ The tobacco control program in India has been successful in initiating and sustaining collaborative work, but it remains highly contested, complex and challenging. Historically, India has been a cultivator and exporter of tobacco, and this crop remains a source of individual and government revenues. The creation of the NTCP exposed conflicting sectoral goals and institutional mandates for other ministries, like the Ministry of Commerce and small-scale cottage industries, under which bidi is covered, or Agricultural Ministries with research institutes for tobacco, thus leading to policy incoherence. In such complex policy environments sharing responsibilities and implementing collaborative, multisectoral action becomes challenging.

 As a result, efforts until now have largely focussed on curbing the demand side of tobacco, while much more work on the supply side is required. However, there have been thinking and early action especially on behalf of MoHFW, in seeking collaboration with Tobacco Research Institutes and Ministry of Agriculture to address economically viable alternate crops,^45^ support by technical support groups to organize expert consultations.^46^ Initiating such action would also require concentrated efforts from Ministry of Agriculture and other stakeholder departments to enable wholistic planning from providing subsidies for alternate crops to strengthening its supply chain and distribution process, engaging the farmers at every step.^47^

 The second interlinked challenge is the influence of the tobacco industry, which is well-documented globally.^48-50^ Tobacco industries are multi-national corporations, powerful, richly resourced, with experienced lobbying tactics. They have innovated in marketing newer tobacco products to circumvent tobacco legislation and have hindered the processes of policy adoption and implementation of tobacco control. Their role has been documented in influencing the implementation of pictorial warnings on tobacco products in India.^51^ The use of legal instruments and litigation has been the most successful mechanism to limit industry interference. Hence, the presence of a strong legal framework and the mobilization of political and societal actors has been central to the tobacco control program in India.

 There is now a need for a ‘second generation’ of tobacco control in India that is being referred to as ‘COTPA-II.’ This would require strengthening the legal foundation, as there are current gaps in the law for point of sale (vendor licensing) and advertisement bans, non-sensitivity to smokeless forms of tobacco, and the number and smaller amount of fines charged. The COTPA amendment was opened by the Ministry of Health in 2015, which followed a pre-legislative consultation process and was available in the public domain. But, later, the amendment bill was withdrawn to re-look at the draft provisions as it faced resistance from industry representatives, certain farmer groups and retailers. This opposition was demonstrated widely across the country and captured by various media outlets. In addition, the next stage of policy reform has to address the supply side of tobacco, addressing core issues of industry interference and alternate cultivation promotion. However, sustainability of funding for advocacy efforts and continued research would be imperative to move into the next phase of reforms.

###  Strengths and Limitations

 This multisectoral case study research focussed on tobacco control policy in India, highlighting the key contextual features, actors, processes and drivers, and providing a comprehensive policy landscape. Moreover, the study interviewed actors across different government sectors, civil societies and national-subnational levels and applied a framework that takes into account the role of political dimensions, role and interconnected relationship of key actors in the tobacco policy environment. However, there are certain limitations. First, our study at the state level focuses on a single case, Karnataka, which is a tobacco-producing state, and there may be significant variation in terms of political regime, economic growth models, the vibrancy of civil societies and overall tobacco landscape; hence, we caution adapting the findings for other multisectoral policies and in other contexts. Second, there is a temporal gap, as major laws and policies began as early as 2003, thus exact sequential details and some points in the trajectory may not have been captured through interviews. Additionally, there have been almost two decades of evolution of these policies, thus limiting the respondents recall of exact events, especially during the early phases and development of tobacco control. Third, we were unable to secure interviews with certain selected government officials, and hence some of their perspectives were not captured, which may have limited our understanding of the facts and interpretation of perceptions around the evolution of tobacco policy. Finally, we did not engage with the tobacco industry and representatives of farming communities and cooperatives, as this would have substantially increased the scope of the study. Thus, the findings might not fully incorporate potentially important perspectives, such as other viewpoints of these groups of actors. However, the respondents of the study did mention the roles played by these stakeholders, but their perspectives are absent in the study, and future studies can include these stakeholders in their research.

## Conclusion

 Findings from this analysis highlight the complex, dynamic, constantly evolving and multi-faceted nature of multisectoral health policy processes. It sheds light on the enablers for policy development and adoption, including the need for collaborative action, mobilization of legal and political frameworks, and social advocacy to bring about intended policy change. The process of collaboration, however, is not a panacea, and its associated challenges and paradoxes that need to be understood and contextualized. Insights from this analysis may help practitioners and researchers understand the policy process in the case of a multisectoral policy, especially in an LMIC context. The analysis also shares how different stakeholder groups can engage and influence policy-making and the process of adoption. The findings also suggest that in a multisectoral policy, a whole-of-society approach or the engagement of a whole system approach rooted in realizing the need for a joint action is necessary to propel the collaborative process. The critical challenges identified here further enhance our understanding and contribute towards the generation of knowledge in terms of ‘what needs to be done’ to advance such policies in the context of sustainable development, where the nature of problems and their solutions require working across boundaries to establish collaboration.

## Acknowledgements

 We would like to acknowledge Dr. Pragati Hebbar, PhD scholar and DBT/Wellcome Trust India Alliance Early Career Fellow at the Institute of Public Health, Bengaluru for her suggestions that helped in improving the manuscript.

## Ethical issues

 The ethics approval for the study was obtained from the Institutional Ethics Committee at the Institute of Public Health, Bengaluru (IEC-ER/01/2018) and the Faculty of Medicine, McGill University (A05-E24-18B).

## Competing interests

 Authors declare that they have no competing interests.

## Authors’ contributions

 SM, SVB, UB, SL, and AM were engaged in conception and design of the study. SM led the development of study instruments, led analysis, and wrote the first draft of the manuscript. SM, SVB, UB, SL, and AM analysed and interpreted the data. SVB, UB, SL, and AM engaged in providing important intellectual content, editing and approval of the manuscript. All authors read, edited and approved the final manuscript.

## Funding

 SM received doctoral funding from The Common Threads through the Commonwealth Canadian Queen Elizabeth II Diamond Jubilee Scholarship Program (CTC-QES Program) at McGill University & the Fonds de Recherche en Santé du Québec. UB is supported by DBT/Welcome Trust India Alliance fellowship (IA/CPHI/17/1/503346). Funders had no role in design, implementation and interpretation of study results or writing this manuscript.

## Supplementary files

Supplementary file 1 contains Tables S1-S3.
