# Supplementary material for: Policy Processes in Multisectoral Tobacco Control in India: The Role of Institutional Architecture, Political Engagement and Legal Interventions
Source: Int J Health Policy Manag. 2021 Jul 14;11(9):1703–14. doi: 10.34172/ijhpm.2021.66 (PMC9808220; doi:10.34172/ijhpm.2021.66)
Supplement: Supplementary file 1 — contains Tables S1-S3. [file ijhpm-11-1703-s001.pdf]

Article title: Policy Processes in Multisectoral Tobacco Control in India: The Role of Institutional Architecture, Political Engagement and Legal Interventions

Journal name: International Journal of Health Policy and Management (IJHPM)

Authors' information: Shinjini Mondal[1]*, Sara Van Belle[2], Upendra Bhojani[3], Susan Law[4,5], Antonia Maioni[6]

[1]Department of Family Medicine, McGill University, Montreal, QC, Canada.

[2]Department of Public Health, Health Policy Unit, Institute of Tropical Medicine, Antwerp, Belgium.

[3]Institute of Public Health, Bengaluru, Karnataka, India.

[4]Institute for Better Health, Trillium Health Partners, Mississauga, ON, Canada.

[5]Institute of Health Policy, Management and Evaluation, University of Toronto, Toronto, ON, Canada.

[6]Department of Political Science, Faculty of Arts, McGill University, Montreal, QC, Canada.

(*corresponding author: shinjini.mondal@mail.mcgill.ca)

Supplementary file 1

Table S1. Thematic Categories and Themes

Broad Thematic Category	Themes
History, evolution & context	Context of initiation (national/state/international)<br>Role of acts/policies<br>Participation/engagement of key organizations/individuals in shaping<br>Evolution/changes over the last decade
Description of role & engagement	Role in the tobacco control program<br>Reason for engagement<br>Engagement process, key events
Critical points/junctions	Most critical points that shaped the policy formulation
Current policy status & impact	Status of current policy (national/state)<br>Impact at national/state level<br>Role of Key partners/institutions/individuals/organization
Success/achievements	Description of success at national/state level<br>Reason behind those success
Challenges	Description of challenges at national/state level<br>Reason behind those challenges
Suggestions/strengthening	Strengthening of program/changes<br>Future challenges

Table S2. Summary Table of National Documents Reviewed

Policy/Act/circular	Sections/details of the policy	Details
National level		
The Cigarettes and Other Tobacco Products (Prohibition of Advertisement and Regulation of Trade and Commerce, Production, Supply and Distribution) Act, 2003 (Act No. 34 of 2003) (COTPA)	- The first provisions of COTPA entered into force on May 1, 2004. These provisions included Sections 1-5, 6(a), 12(1)(b), 12(2), 13(1)(b), 13(2), 14, 16, 19, 21-31<br>- Sections 12(1)(a), 13(1)(a), 15, 17, 18, 32, and 33 took effect on July 30, 2009<br>- Section 6(b) regarding the sale of cigarettes around educational institutions, took in effect from 18, 2009	- Principal law governing tobacco control in India<br>- Restrictions on smoking in public places; advertising, promotion and sponsorship; sales to minors; packaging and labelling; and enforcement and penalties
The Food Safety & Standards Act 2006	- Authorises State Commissioner of Food Safety to prohibit, the manufacture, storage, distribution, or sale of any article of food, in the interest of public health	-Several states utilized this authority to ban certain forms of smokeless tobacco.

The Food Safety and Standards (Prohibition and Restrictions on Sales) Regulations, 2011	-Prohibits tobacco and nicotine from being used in any food products.	Courts in several states have relied on this provision to impose bans on the manufacture, distribution, and sale of "gutka" or "pan masala."
**Tobacco Packaging & Labelling**<br>- Cigarettes and Other Tobacco Products Packaging and Labelling (Amendment) Rules, 2008.<br><br>1) G.S.R. 182(E) announces the Cigarettes and Other Tobacco Products (Packaging and Labelling) Rules, 2008-March 15, 2008<br><br>2) G.S.R. 305(E) announces the Cigarettes and Other Tobacco Products (Packaging and Labelling) Amendment Rules, 2009-May 3,2009<br><br>5) G.S.R. 1866(E)- 30 July, 2009	-contains substitute language regarding health warnings on retail packaging, requiring warnings to be printed on external packaging such as cartons.<br><br>-issued under COTPA, the rules specify components of the health warnings (i.e., content, size, rotation, etc.), but various provisions in subsequent rules replace certain language in the 2008 regulations.<br><br>-Contains substitute language for the definition of "package" and for the location of the health warning. It also deletes the requirement that the warning be located on both sides of box and pouch type packs.<br><br>-It authorizes certain officers, in addition to those already designated in COTPA, to carry out the entry, search, and seizure provisions in COTPA Section 12 (with respect to any violation of the Act) and Section 13 (with respect to violations of tobacco product packaging and advertising).	

6) G.S.R. 680(E)- 15 September, 2009	- It adds to the listing of additional persons authorized to collect fines for the violation of specified smoke free rules.	
7) G.S.R. 985(E) announces the Cigarettes and Other Tobacco Products (Packaging and Labelling) Amendment Rules, 2010- 20 December, 2010	- this notification contains substitute language on the issue of rotation, requiring that health warnings be rotated every 24 months instead of one year. The rule also re-establishes the May 2009 health warnings, ensuring that pictures of a lung x-ray and diseased lungs continue to be displayed on smoked tobacco product packages and a picture of a scorpion continues to be displayed on smokeless tobacco product packages	
8) G.S.R. 570(E), the Cigarettes and other Tobacco Products (Packaging and Labelling) Amendment Rules, 2011, amends a rule announced in G.S.R. 182(E) – 26 July, 2011	- regarding the languages in which the health warnings are written and updates the components of the health warning	
9) G.S.R. 417(E) announces the Cigarettes and other Tobacco Products (Packaging and Labelling) Amendment Rules, 2011- 27 May 2011	-the rules establish new graphic health warnings for packages of smoked and smokeless forms of tobacco. The rules also increase the number of warnings for smoked tobacco products from two to four, and increases the number of warnings for smokeless tobacco products from one to four.	

10) G.S.R. 724(E) announces the Cigarettes and other Tobacco Products (Packaging and Labelling) Amendment Rules, 2012- 27 September, 2012<br><br>11) G.S.R. 724(E) announces the Cigarettes and other Tobacco Products (Packaging and Labelling) Amendment Rules, 2012- 24 September 2015<br><br>12) 727(E) announces the Cigarettes and other Tobacco Products (Packaging and Labelling) Amendment Rules, 2014. 15 October, 2014<br><br>13) G.S.R. 292(E) announces the Cigarettes and other Tobacco Products (Packaging and Labelling) Amendment Rules, 2017.<br>24 March, 2017<br><br>14) G.S.R. 283(E) announces the Cigarettes and other Tobacco Products (Packaging and Labelling) Amendment Rules, 2018.. 26 March, 2016<br><br>15) G.S.R. 331(E) announces the Cigarettes and other Tobacco Products (Packaging and Labelling) Second Amendment Rules, 2018.<br>3 April, 2018	- The Rules establish new health warnings for tobacco product packaging, effective April 1, 2013<br><br>-Called for new health warnings covering 85% of the front and back of tobacco product packaging<br><br>- The Rules establish, among other items, new health warnings to cover 85% of the front and back of tobacco product packaging.<br><br>- The rules establish that the second of the two health warnings contained in G.S.R. 727(E) is to be used on product packaging beginning April 1, 2017<br><br>- The amended rules establish that the existing health warnings are to remain in rotation until August 31, 2018<br><br>- The amended rules establish the next round of pictorial health warnings, which are required to appear on tobacco product packaging beginning on September 1, 2018. The new health warnings also include a quit-line phone number.	
**Tobacco Advertising, Promotion and Sponsorship**		

Cable Television Networks (Regulation) Act, 1995 (CTNA) and Cable Television Networks (amendment) Rule 2009<br><br>- G.S.R. 345(E)<br><br>G.S.R. 619(E)<br><br>G.S.R. 786(E)<br><br>G.S.R. 708(E)	- Prohibits direct advertising of cigarettes or tobacco products<br><br>- amends the Rules by substituting new provisions on point of sale advertising and adding a definition of indirect advertising<br>- provides additional point of sale rules<br>- establishes rules for television and film and print and outdoor media<br>- updates the rules for television and film	- Implementing rules prohibit direct advertising of tobacco products on Indian cable networks, but permit the indirect advertising of such products under certain circumstances. (Acc to July 2010 Ministry of Information and Broadcasting Directive).<br>-CTNA does not regulate international cable television networks.
National Tobacco Control Program- Operational Guidelines 2013/2015	Government of India launched the National Tobacco Control Program (NTCP) in the year 2007-08, with the aim to<br>create awareness about the harmful effects of tobacco consumption,<br>(ii) reduce the production and supply of tobacco products,<br>(iii) ensure effective implementation of the provisions under “The Cigarettes and Other Tobacco Products (Prohibition of Advertisement and Regulation of Trade and Commerce, Production, Supply and Distribution) Act, 2003” (COTPA) (iv) help the people quit tobacco use, and<br>(v) facilitate implementation of strategies for prevention and control of tobacco advocated by WHO Framework Convention of Tobacco Control	The main thrust areas for the NTCP are as under:<br>Training of health and social workers, NGOs, school teachers, and enforcement officers;<br>(ii) Information, education, and communication (IEC) activities;<br>(iii) School programs;<br>(iv) Monitoring of tobacco control laws;<br>(v) Coordination with Panchayati Raj Institutions for village level activities;<br>(vi) Setting-up and strengthening of cessation facilities including provision of pharmacological treatment facilities at district level

Table S3. summary Table of State Level Documents Reviewed

Policy/Act/circular	Sections/details of the policy	Details
**State level**		

Circular issued by<br>Director general of police, and director general of Karnataka fire and emergency services<br>NO GBC (1) 190/2014.<br>Dated: 22-09-2014	Addressed to:<br>1) All the chief fire officers.<br>2) All the regional fire officers.<br>3) All the district fire officers.<br>4) All the fire station officers.<br><br>Include Section 4 of COTPA into the fire safety code for buildings in Karnataka.	Section 4 of COTPA into fire safety code for buildings.<br><br>No Smoking Board as specified in schedule II of COTPA to be displayed at entrance of public place.
Order Issues by:<br>Member Secretary State Anti-Tobacco Cell Bangalore<br>DHS/Tambaku/ 27-11-12.<br><br>Dated: 5-1-2012	Addressed to:<br>District Health and Family welfare officers and Member secretary District Anti - Tobacco Cell.<br>Making School Environments Tobacco free	Section 6 of COTPA<br>Tobacco free schools
Circular issued by Commissioner Department of Public Education<br>C4(6) Sa.Sta.Dhu.Ni 01-2013-14.<br><br>Dated: 5-11-2013.	Addressed to: All Deputy directors Department of Public Education.<br><br>Holding a state level awareness program about ill effects of Tobacco to students and teachers.	Section 6 of COTPA
Circular issued by Under secretary<br>Transport Department.<br><br>Sam :Sa Ri E Sa Sam E 2014<br><br>Dated: 13-2-2014	Circular issued by Under secretary Transport Department.<br><br>Sam :Sa Ri E Sa Sam E 2014<br><br>Dated: 13-2-2014	Section 4 of COTPA
Circular issued by<br>Special Officer and Ex Officio<br>Under-secretary Education Department. (Planning)<br>C4(6) Sa. Sta.Dhu. Ni/36/ 2008-2009<br><br>Dated: 24-4-2014.	Addressed to: Deputy Director, Public Education Department all districts.<br><br>Display of no selling to tobacco within 100 Yards of the schools/colleges and educational institutes.	Refers to COTPA Section-6<br>Education department and police department to work together on tobacco free schools.
Circular issued by<br>Principal Secretary Health	Addressed to: The Editor Karnataka Gazette Bangalore	Refers to<br>Section 4 of COTPA

and Family Welfare Department.<br>HFW 461 CGE 2013<br><br>Dated: 29-5-2014	Copy to: Chief Secretary Govt. of Karnataka.<br><br>Prohibiting smoking and consumption of all forms of tobacco products in Government Offices and buildings.	Section 21 of COTPA
Circular issued by<br>Director (Panchayat Raj-2), Ex Officio<br>Joint-secretary Rural Development and Panchayath Raj.<br><br>Gra A Pa 38 2014<br><br>Dated: 7-8-2014	Addressed to:<br>To all District Commissioners,<br>To ALL CEO Zilla Panchayath, to bring this circular into Taluk panchayat and Graam Panchayath Notice.<br>TO Web site and Karnataka Development.<br><br>Guidelines for Implementation of COTPA.	Section 4, Section 5, Section 6 and Section 7 of COTPA.
Circular issued by Commissioner Department of Public Education<br>C4(6) Sa.Sta.Dhu.Ni-36/2008-09<br><br>Dated: 18-3-2015.	Addressed to: All Deputy directors Department of Public Education.<br><br>COTPA awareness among students and teachers.	Section 4 of COTPA
Circular issued by<br>Commissioner of exercise Government of Karnataka<br>ECI/26/JIML/2014-15<br><br>Dated: 25-3-2015	All Deputy commissioners Exercise department all districts.<br><br>Implementation of COTPA Act 2003.	Section 4 of COTPA
Order Issued by:<br>Principal Secretary<br>Administrative Department<br>Govt. of Karnataka.<br>OE 35 PCB 2013<br><br>Dated: 22-6-2015.	Addressed to:<br>Joint Director and Inspector General of Police, Nrupatunga Road, Bangalore.<br><br>Principal Secretary, Primary and Secondary Education, Multi storied building, Bangalore.	Inter sectoral action for COTPA Implementation.<br><br>As per the Letter written by, Member High Power committee on Tobacco control. “Tobacco Free school” or “Tobacco free educational institute.

	Principal Secretary, Health and Family Welfare Department, Vikasa Soudha, Bangalore.<br><br>Principal Secretary,<br>Urban Development<br>Vikasa Soudha, Bangalore.<br><br>Commissioner<br>Health and Family Welfare Department, Anandrao Circle<br>Bangalore.<br><br>Inter sectoral action for effective Implementation of COTPA 2003.	
Circular issued by<br>Principal Secretary Department of Information Technology, Bio technology and Science and technology<br><br>Dated: 26-10-2015	Addressed to: Sri Biren Ghosh ABAI, HSR layout Bangalore -02.<br><br>COTPA Implementation in IT -BT Sector	Section 4 of COTPA
Issued by:<br>Compliance Officer (COTPA) Deputy Commissioner of Police (Crime) Bangalore.<br>63/ccrb/review/2015<br><br>Dated: 31-8-2017	Issued by:<br>Compliance Officer (COTPA) Deputy Commissioner of Police (Crime) Bangalore.<br>63/ccrb/review/2015<br><br>Dated: 31-8-2017	Implementation of smoke free laws
E cigarette Ban<br><br>State govt hereby prohibits the sale, manufacture, distribution, trade, import and advertisement of ENDS its parts and	Addressed to:<br>To All additional chief secretaries/ Principal Secretary/ secretaries of the government,<br>All Police Commissioners,<br>All District Deputy Commissioners,<br>All District Superintendents of	Drug and Cosmetic act 1940.<br>Food safety and standard act 2006.<br><br>Use of nicotine in food products and consumption by public is banned under food safety and tandard act 2006. r/w notification NO: F.NO2-

components in any shape or size of cartridges containing nicotine in the interest of public.<br><br>Circular issued by Deputy secretary to Government Health and Family Welfare Department & Nodal Officer High power committee for tobacco control<br><br>No HFW/126/CGE/2016.<br><br>Dated: 15-6-2016.	police.<br>ALL CEO Zilla Panchayath,	15015/13/2010, Dated 1/8/2001 and under para 2, 3, 4 of food safety and standards regulation 2011.<br><br>The use of nicotine in food products preparation i.e use of this chemical in any form is banned in India under food safety and standard act 2006. Nicotine is allowed as an aid for de-addiction in nicotine replacement therapy under drugs and cosmetics act 1940.
